# Oral *Candida* colonization and virulence in children with orofacial clefts: a cross-sectional study

**DOI:** 10.1186/s12903-026-09253-y

**Published:** 2026-07-18

**Authors:** Nouran N. Alazhary, Eman A. Omran, Nagwan E. S. Youssef, Abdelfattah H. Abdelfattah, Aly M. Atteya

**Affiliations:** 1 Department of Microbiology. High Institute of Public Health, Alexandria University, Alexandria, Egypt; 2 Department of Maxillofacial and Plastic Surgery, Faculty of Dentistry, Alexandria University, Alexandria, Egypt

**Keywords:** *Candida* species, cleft palate, cleft lip and palate, virulence, phospholipase, hemolysin

## Abstract

**Background:**

Orofacial clefts (OFCs) are congenital anomalies which include cleft palate (CP) and cleft lip and palate (CLP). These anatomical mal-fusion of the palate may favor the abnormal overgrowth of oral microorganisms, such as *Candida* spp. This study aimed to address the colonization rate of *Candida* spp. found in the oral cavity of children with OFCs compared to children with NP. Since *Candida* spp. vary in their pathogenicity and response to antifungals, determining the oral carriage rate of *Candida* spp. and assessing their virulence factors is important to determine the need for administering antifungal therapy to OFC patients and identify if specific types of clefts are more prone to colonization than others. Moreover, determining whether enzyme production by different *Candida* spp. is accentuated among OFCs would help understand the pathogenesis of oral candidiasis among this group of patients.

**Methods:**

A comparative cross-sectional study included 40 children with OFCs and 40 others with normal palate (NP). Data on oral hygiene and feeding habits was collected. Oral swabs were obtained from both study groups and were cultured for *Candida* species counting and presumptive identification using CHROM agar. Phospholipase and hemolysin enzymatic activities were determined for all *Candida* species as determinants of virulence.

**Results:**

Positive *Candida* colonization was found in 80% of OFCs children and 57.5% of NP children, with *C. albicans* being the predominant species in both study groups. The median *Candida* count was higher in OFCs children than in NP children (*p* < 0.001). *C. albicans* was the most prevalent species in patients with bilateral cleft lip and palate (CLP) (75% *n* = 15), soft and hard CP (*n* = 5, 38.5%), unilateral CLP (*n* = 3, 30%), and soft CP (*n* = 1, 11.1%), respectively. There was no significant association between colonization and pathogenicity using hemolysin or phospholipase activity with any particular species or study group (*p* > 0.05).

**Conclusion:**

This study demonstrates differences in oral *Candida* colonization between children with OFCs and those with NPs, with variation in colonization burden and species distribution between the two groups. The assessment of key virulence factors did not reveal group- or species-specific associations. These findings highlight the importance of comprehensive assessment of oral *Candida* colonization in pediatric populations with OFCs.

## Introduction

Orofacial clefts (OFCs) are common birth defects characterized by incomplete fusion of the lip and/or palate during fetal development. They can range from small notches to complete separations, affecting the appearance, speech, hearing, breathing and feeding abilities. Although the exact causes are not fully understood, a combination of genetic and maternal factors is thought to play a role [1]. Treatment involves a multidisciplinary approach, including surgery, dental care, speech therapy and medical support, to help individuals with OFCs improve their quality of life [2]. Globally, the World Health Organization (WHO) reported that the overall prevalence of the OFCs was 1 per 1,000 births [3]. In Egypt, it was estimated to be 4 per 10,000 births [4].

*Candida* is the most common fungal colonizer found in the oral cavity [5]. It is part of the body’s normal flora and is found on most mucocutaneous surfaces such as the mouth, gastrointestinal tract, eyes, ears, and nose with *Candida albicans* (*C. albicans*) being the most common species [6]. Under certain circumstances, *Candida* colonization can lead to the development of oral thrush, which is a fungal infection of the mouth and throat caused by overgrowth of *Candida* species [7]. This can be particularly challenging for patients with clefts, who may already have difficulty with natural suction and swallowing, as well as decreased salivary flow and an impaired immune system [8]. Oral thrush can cause pain and discomfort, making eating and drinking more difficult [7]. Additionally, *Candida* colonization can contribute to the development of dental caries and periodontal disease, both of which can worsen oral health. *Candida* can also cause chronic inflammation of the oral tissues, which can contribute to the development of scar tissue and further complicate cleft repair surgeries [9, 10].

Extracellular hydrolytic enzymes produced by *Candida* play a vital role in its pathogenicity. These enzymes facilitate tissue penetration, adhesion and consequently invasion into the host [11, 12]. Phospholipases are a group of the most important hydrolytic enzymes produced by *Candida* [11]. They disrupt the phospholipids present in the cell membrane causing cell lysis, adhesion and infection [13]. Hemolysin secretion is another virulence factor, which is followed by iron uptake that promotes hyphal invasion in cases of disseminated candidiasis [11, 13, 14].

The oral flora of children with OFCs is likely to undergo significant alterations due to the anatomical disruption at the interface between the oral cavity and the nasopharyngeal space [15]. This structural discontinuity can create an environment that favors microbial imbalance and increased susceptibility to colonization by opportunistic organisms [16]. Previous studies have reported that individuals with OFCs exhibit a markedly higher rate of *Candida* colonization compared with healthy controls, highlighting the clinical importance of monitoring fungal presence in this population [17, 18]. In light of these findings, the present study was designed to investigate different patterns of oral *Candida* colonization in children with and without OFCs. In addition, this work aims to conduct an in-depth in vitro assessment of two key virulence factors—phospholipase and hemolysin activity— both of which play critical roles in the pathogenic potential of *Candida* species. By evaluating these enzymatic activities, the study seeks to provide a clearer understanding of whether children with OFCs harbor strains with enhanced virulence, which may contribute to increased risk of oral or systemic fungal infections. Ultimately, this research endeavors to offer valuable insights into the microbiological profile of children with clefts, support early detection of pathogenic colonization and to help guide future preventive or therapeutic strategies tailored to this vulnerable population.

Accordingly, this comparative cross-sectional study aims to evaluate oral *Candida* colonization in patients with OFCs in comparison with a non-affected control group (children with NP). The null hypothesis (H₀) postulates that there are no statistically significant differences in oral *Candida* colonization between patients with OFCs and the control group.

## Materials and methods

This comparative cross-sectional study was conducted in accordance with the principles outlined in the Declaration of Helsinki and received approval from the Institutional Review Board (IRB) committee, High Institute of Public Health; IRB number 00013692, serial number AU092388158. Due to the comparative cross-sectional nature of the study, the clinical trial number was not provided. Sample size calculation was performed using G*Power software (version 3.1.9.6). Based on a previous study, the asymptomatic oral carriage rate of *Candida* spp. was 63.3% in patients with CP and 18.3% in those with NP [17]. The effect size (Cohen’s h) calculated from these proportions was 0.94, indicating a large effect size. Using a two-tailed test for comparison between two independent proportions, with a significance level (α) of 0.05 and statistical power of 80%, the minimum required sample size was estimated to be 50 participants (25 per group). To enhance the robustness of the results, a total of 80 participants (40 per group) were ultimately included.

### Study participants

A total of 80 children less than 5 years of age were included in the present study. They were allocated in 2 groups: the first group included 40 children with OFCs (22 children with CP and 18 children with CLP) and the second group included 40 cross-matched healthy children with NP as the control group. Both groups were matched according to their age (age less than 5 years) and sex. They were admitted and assessed by the Maxillofacial and Plastic Surgery Clinic of Alexandria University Dentistry Hospital. All children with comorbidities, diabetes mellitus, or those taking antibiotics and/or antifungals in the last 4 weeks were excluded from the study. Prior to participation, written informed consent was obtained from each participant’s parent or legal guardian. The consent process involved providing detailed information about the study’s purpose and procedures. The parents or legal guardians were given the opportunity to ask questions and were assured that their child’s participation was voluntary and could be withdrawn at any time without any consequences.

### Data collection

A structured interview questionnaire was completed for each participant enrolled in this study. The form included patient’s sociodemographic data as name, age and sex. A detailed history of lactation was also obtained, covering both the mode of feeding (breastfeeding, bottle-feeding, mixed) and the duration of lactation. Additionally, the questionnaire addressed several oral habits and hygiene-related factors that may impact oral health status. These included the frequency of dental visits, the use of a pacifier, tooth-brushing practices and other habits such as finger/hand sucking. Caregivers were also asked about the child’s dietary behaviour, including the frequency of consuming sweet foods and the number of meals taken per day. Other functional habits, such as mouth breathing, the presence of halitosis or any difficulty in swallowing (dysphagia), were also documented. Additionally, a thorough clinical examination was subsequently conducted for every patient to evaluate the type of cleft palate present. The oral mucosa was carefully inspected to assess the presence or absence of hyperemia, as well as any observable white patches suggestive of oral candidal colonization or other mucosal alterations.

### Sample collection

A single mouth swab was collected aseptically from each patient fulfilling the predetermined criteria. Sampling was performed by one examiner to maintain uniformity. A sterile cotton swab was moistened with sterile normal saline and then gently wiped along the dorsum of the tongue and the buccal and palatal mucosae with a rotating motion [19]. The culture swabs were withdrawn and immediately placed in a sterile empty tube. All swabs were labelled and promptly transported to the laboratory to be cultured.

### *Candida* species isolation and identification

The cotton swab obtained was inoculated across the surface of a plate of chromogenic agar medium for *Candida* isolation (CHROMagar *Candida*; CHROMagar, Paris, France) containing 0.5 g/L chloramphenicol and was labelled and incubated (using incubator; Memmert, Schwabach, Germany) aerobically at 37 °C for 48 h. At the end of the incubation period, the differently colored colonies were identified according to the manufacturer’s instructions as listed in Table 1. The absence of colonies on CHROM agar was reported as “no growth”.

**Table 1:** Colour appearance of different *Candida* species on CHROMagar media [20]

Microorganism	Typical colony appearance
*C. albicans*	Green
*C. tropicalis*	Metallic blue
*C. krusei*	Pink, fuzzy
*C. kefyr*,* C.glabrata*	Mauve-brown
Other species	White to mauve

### Counting colonies

The number of *Candida* colonies which appeared on the CHROM agar were counted using colony counter after incubation at 37 °C for 48 h and expressed as colony-forming unit (CFU/mL). Mixed species of *Candida* identified by the colony colors on the CHROM agar *Candida* plates were also counted.

### Determination of virulence factors

Before performing the hemolysin and phospholipase tests, colonies were subcultured on Sabouraud dextrose agar (SDA) (Oxoid; Thermo Fisher Scientific, Basingstoke, UK) supplemented with chloramphenicol 0.5 g/L. Plates were prepared and each isolated colony from the CHROM agar was subcultured on SDA plates. In the case of mixed growth, a colony from each colour was streaked on a separate plate to obtain a pure and fresh culture. Each plate was labelled and incubated aerobically at 37 °C for 18–20 h.

Hemolysin production of the isolates was evaluated using the plate assay method described by Manns et al. [21]. The blood agar medium was prepared and poured in 9 cm petri dishes. A yeast suspension (5 µL) equal to McFarland 0.5 turbidity standard was prepared and a 10 µL of the suspension was spot inoculated aseptically on the blood agar medium and incubated at 37 °C for 48 h [22]. The presence of a distinct translucent halo around the inoculum site, viewed with transmitted light, indicated positive hemolytic activity [23]. The diameters of the zones of lysis and the colony were measured using a hemolytic index (HI) to represent the intensity of hemolysin enzyme production by different *Candida* spp. HI was calculated in terms of ratio of the diameter of the colony to the total diameter of the colony plus the zone of precipitation. Results were classified into five categories based on their HI score. HI ≤ 0.69 indicated high hemolytic activity; HI with 0.70–0.79 referred to moderate hemolytic activity; HI = 0.80–0.89 corresponded to low hemolytic activity; HI = 0.90–0.99 meant very low hemolytic activity and HI = 1 indicated negative hemolytic activity.

The phospholipase production of the isolates was assayed using the egg yolk agar plate method described by Price et al. [24]. The egg yolk agar medium was prepared and poured into 9 cm Petri dishes. A yeast suspension (5 µL) equal to McFarland 0.5 turbidity standard was prepared and 10 µL of the suspension was spot inoculated aseptically on the medium prepared and incubated at 37 °C for 48 h. The appearance of a dense white precipitation zone around the colonies indicated a positive phospholipase activity. Phospholipase activity (Pz Value) was then measured in terms of the ratio of the diameter of the colony to the total diameter of the colony plus the zone of precipitation and further classified into five categories [24]. Pz ≤ 0.69 indicated high phospholipase activity; Pz with 0.70–0.79 referred to moderate phospholipase activity; Pz = 0.80–0.89 corresponded to low phospholipase activity; Pz = 0.90–0.99 meant very low phospholipase activity and Pz = 1 indicated negative phospholipase activity [25].

### Statistical analysis

The data underwent extraction, revision, coding and input into statistical software (IBM SPSS Statistics for Windows, version 25; IBM Corp., Armonk, NY, USA). Qualitative data were summarized using frequency and percentage, whereas quantitative data. Normal distribution of the data was assessed using the Kolmogorov-Smirnov test. As the data were found to follow a non-normal distribution, non-parametric tests, specifically the Mann-Whitney U test, were employed for comparing quantitative variables. The Chi-squared test (χ²) was utilized to assess the association between categorical variables. In cases where the Chi-squared test was invalid, either the Fisher’s exact test or Montecarlo exact test was employed. Additionally, Spearman correlation coefficient (r) was calculated to examine the association between continuous variables. All statistical analyses were conducted using two-tailed tests, with a predetermined significance level of *p (< 0.05).*

## Results

This comparative cross-sectional study was carried out over six months. A total of 40 children with OFCs were enrolled in the study as well as another 40 children with NP. The mean age of children with OFCs was 12 [± 10.9] months, while that for children with NP was 16.9 [± 12.31] months. The mean weights of children with OFCs and those with NP were 8 ± 3 and 9 ± 4 kg, respectively. Regarding sex, males accounted for 57.5% (*n* = 23) and females for 42.5% (*n* = 17) in both studied groups.

### Oral *Candida* colonization and sociodemographic data, oral habits and oral hygiene behaviour

**Fig. 1:**
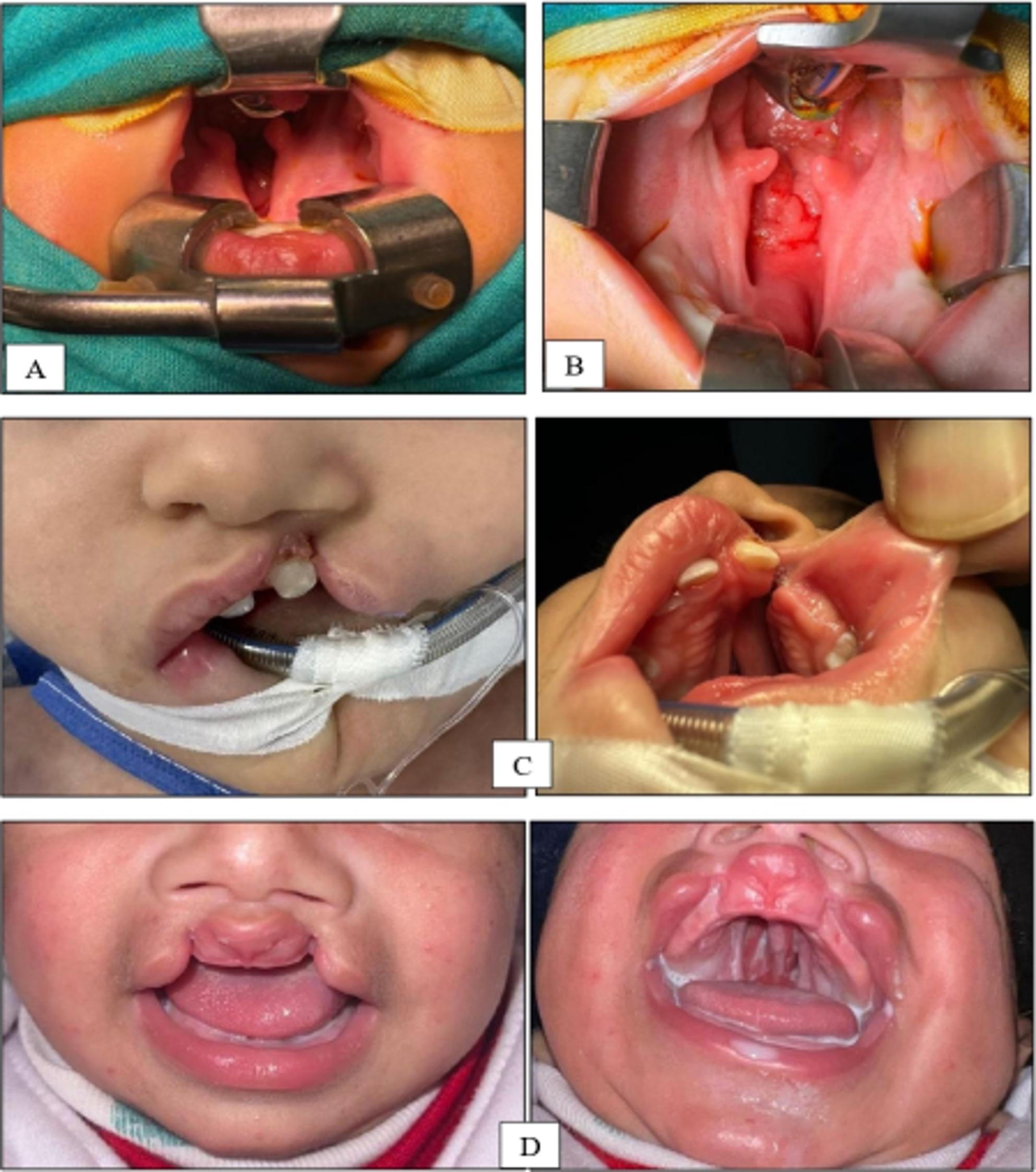
Different types of clefts according to Veau classification of orofacial clefts. **A** Veau I: Defects of the soft palate only. **B** Veau II: Defects of the soft and hard palate. **C** Veau III: Complete unilateral cleft, extending from the soft palate to the alveolus, usually involving the lip. **D** Veau IV: Complete bilateral cleft, extending from the soft palate to the alveolus, usually involving the lip

Table 2 highlights the relationship between oral *Candida* colonization and the participants’ sociodemographic data, oral habits and oral hygiene. There was no significant association between *Candida* colonization and any of the studied factors in children with NP (*p* > 0.05). In children with OFCs, *Candida* colonization showed a statistically significant association with the presence of white patches (*p* = 0.044), however, one OFC patient (5%) who had white patches was found to be not colonized. All 100% (*n* = 5) of NP children with white patches were colonized with *Candida* compared to 95% (*n* = 19) in OFC children. Additionally, it was found that 76.2% (*n* = 16) of OFC children with positive *Candida* colonization did not consume sweets (*p* = 0.035) (Fig. 1).

**Table 2:** Distribution of the oral *Candida* colonization in children with orofacial clefts and normal palates according to the children’s sociodemographic data, oral habits and oral hygiene, Alexandria 2023

	Children with OFCs				*p*	Children with NP				*p*
	Colonization		No colonization			Colonization		No colonization		
	No.	%	No.	%		No.	%	No.	%	
Age (months)										
1–6 m	15	93.8	1	6.3	0.255	4	57.1	3	42.9	0.942
7–12 m	9	69.2	4	30.8		6	54.5	5	45.5	
13–18 m	3	60.0	2	40.0		4	50.0	4	50.0	
19 m and older	5	83.3	1	16.7		9	64.3	5	35.7	
Sex										
Male	19	82.6	4	17.4	0.702	13	56.5	10	43.5	0.884
Female	13	76.5	4	23.5		10	58.8	7	41.2	
Weight (kg)										
2–6 kg	8	88.9	1	11.1	0.783	6	60.0	4	40.0	0.917
6.1–8 kg	13	76.5	4	23.5		7	63.6	4	36.4	
> 8 kg	11	78.6	3	21.4		10	52.6	9	47.4	
Hyperemia										
No	25	78.1	7	21.9	0.553	20	55.6	16	44.4	0.624
Yes	7	87.5	1	12.5		3	75.0	1	25.0	
White patches										
No	13	65.0	7	35.0	0.044*	18	51.4	17	48.6	0.061
Yes	19	95.0	1	5.0		5	100.0	0	0.0	
Child lactation (*n* = 68) (Up to 2 years only)										
Bottle	22	81.5	5	18.5	1.000	4	80.0	1	20.0	0.493
Breast	3	75.0	1	25.0		10	52.6	9	47.4	
Both	3	75.0	1	25.0		4	44.4	5	55.6	
Dental follow-up visits										
No follow-up	27	77.1	8	22.9	0.738	23	60.5	15	39.5	0.174
Every 6 months	2	100.0	0	0.0		0	0.0	0	0.0	
Yearly	3	100.0	0	0.0		0	0.0	2	100.0	
History of using pacifier										
No	30	83.3	6	16.7	0.172	19	59.4	13	40.6	0.702
Yes	2	50.0	2	50.0		4	50.0	4	50.0	
Tooth brushing										
No	16	76.2	5	23.8	0.639	8	47.1	9	52.9	0.241
Yes	4	66.7	2	33.3		12	66.7	6	33.3	
Frequency of tooth brushing (*n* = 62)										
Never	16	76.2	5	23.8	0.487	8	47.1	9	52.9	0.574
Once daily	3	75.0	1	25.0		6	60.0	4	40.0	
Twice daily	1	100.0	0	0.0		4	66.7	2	33.3	
Three times	0	0.0	1	100.0		2	100.0	0	0.0	
Thumb sucking										
Never	10	76.9	3	23.1	1.000	13	68.4	6	31.6	0.280
Always	17	81.0	4	19.0		5	38.5	8	61.5	
Occasionally	5	83.3	1	16.7		5	62.5	3	37.5	
Consumption of sweets (*n* = 62)										
Never	16	76.2	5	23.8	0.035*	7	46.7	8	53.3	0.414
Sometimes	4	100.0	0	0.0		11	61.1	7	38.9	
Always	0	0.0	2	100.0		2	100.0	0	0.0	
Number of meals/day (*n* = 62)										
2	1	100.0	0	0.0	0.549	3	60.0	2	40.0	0.891
3	10	83.3	2	16.7		12	60.0	8	40.0	
> 3	9	64.3	5	35.7		5	50.0	5	50.0	
Mouth breathing										
No	13	76.5	4	23.5	0.702	19	61.3	12	38.7	0.368
Yes	19	82.6	4	17.4		4	44.4	5	55.6	
Mouth malodour										
Never	21	77.8	6	22.2	0.700	16	57.1	12	42.9	0.479
Sometimes	11	84.6	2	15.4		7	63.6	4	36.4	
Always	0	0.0	0	0.0		0	0.0	1	100.0	
Choking										
No	11	78.6	3	21.4	1.000	19	55.9	15	44.1	0.686
Yes	21	80.8	5	19.2		4	66.7	2	33.3	

*OFCs* Orofacial clefts, *NP* Normal palate

*Statistically significant at *p* ≤ 0.05

Some risk factors for colonization and symptoms of CP were studied (Table 3). Statistically significant difference (*p* < 0.05) regarding child lactation method was seen as 77.1% of children with OFCs were bottle fed *versus* only 15.2% in NP children. Concerning tooth brushing, only 22.2% of children with OFCs had their tooth brushed, resulting in statistical differences when compared with the NP group (*p* = 0.019). Both mouth breathing and choking were reported more (in 57.5% and 65%, respectively) in OFCs children than in NP children (*p* < 0.05). White patches were statistically prevalent in 50% of OFCs children and in 12.5% of children with NP (*p* < 0.001).

**Table 3:** Distribution of children with orofacial clefts and those with normal palate according to their oral habits and hygiene

Characteristics	Children with OFCs (*n* = 40)		Children with NP (*n* = 40)		Test of significance (Chi-square)	*p*
	No.	%	No.	%		
Child lactation (Up to 2 years only, *n* = 68)						
Bottle	27	77.1	5	15.2	26.795	< 0.001*
Breast	4	11.4	19	57.6		
Both	4	11.4	9	27.3		
Tooth brushing (*n* = 62)						
No	21	77.8	17	42.5	5.480	0.019*
Yes	6	22.2	18	45.0		
Mouth breathing						
No	17	42.5	31	77.5	10.208	0.001*
Yes	23	57.5	9	22.5		
Choking						
No	14	35.0	34	85.0	20.833	< 0.001*
Yes	26	65.0	6	15.0		
White patches						
No	20	50	35	87.5	13.091	< 0.001*
Yes	20	50	5	12.5		

*OFCs* Orofacial clefs, *NP* Normal palate

*Statistically significant at *p* ≤ 0.05

**Fig. 2:**
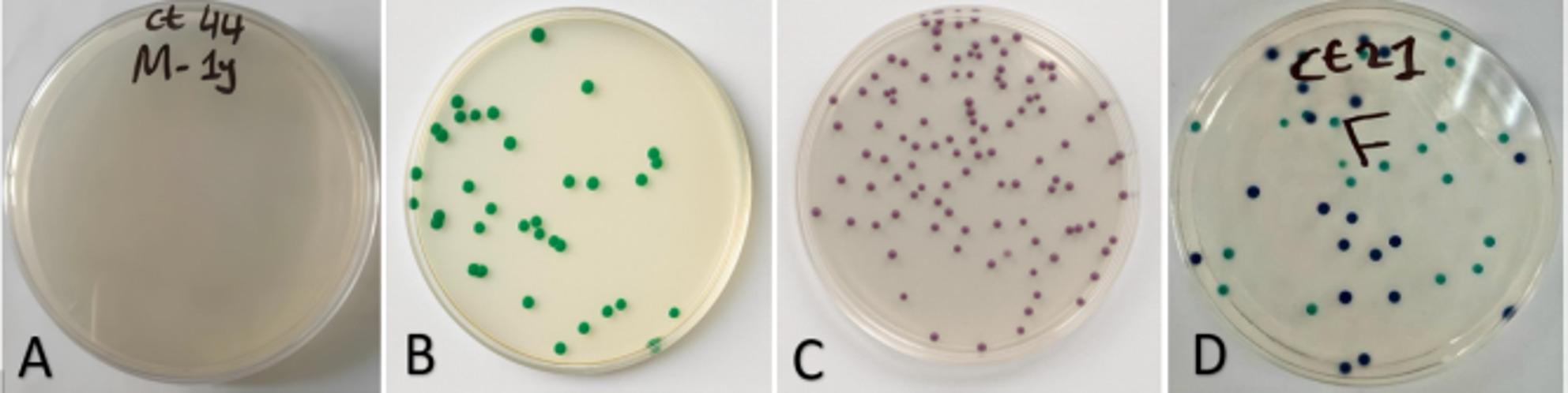
Different plates of CHROM agar showing growth of different *Candida *species. **A** No growth. **B ***C. albicans*. **C** *glabrata*. **D** Mixed growth of *C. albicans* and *C. tropicalis*

As illustrated in Table 4, positive *Candida* cultures accounted for 68.8% of the total samples (*n* = 80). Children with OFCs yielded significantly more positive *Candida* cultures (*n* = 32, 80%) compared to children with NP (*n* = 23, 57.5%), *p* = 0.030. *C. albicans* was the commonest species in both study groups. Children with OFCs had higher rates of mono-species cultures of *C. albicans* and NAC compared to NP children, (37.5% and 35% *versus* 27.5% and 15%, respectively). In contrast, higher rates of mixed *C. albicans* and NAC were more prevalent among children with NPs compared to children with OFCs (15% *versus* 7.5%, respectively). The growth of *Candida* spp (Fig. 2) and the distribution of its species was significantly different between both study groups (*p* = 0.045).

**Table 4:** Distribution of *C. albicans* and NAC mono and mixed species in the oral cavity of children with orofacial clefts and normal palate

	Children with OFCs (*n* = 40)		Children with NP (*n* = 40)		Total (*n* = 80)		*p*
	No.	%	No.	%	No.	%	
*Candida* colonization							0.030*
No growth	8	20.0	17	42.5	25	31.3	
Growth	32	80.0	23	57.5	55	68.8	
*Candida* species							
No growth	8	20.0	17	42.5	25	31.3	0.045*
* C. albicans* mono-species cultures	15	37.5	11	27.5	26	32.5	
Mixed *C. albicans* and NAC	3	7.5	6	15.0	9	11.3	
NAC mono-species cultures	14	35.0	6	15.0	20	25.0	

*NAC* non-*albicans Candida*, *OFCs* Orofacial clefts, *NP* Normal palate

*Statistically significant at *p* ≤ 0.05

Distribution of different *Candida* species in Table 5 showed that, in OFCs patients, *C. tropicalis* (25%) was the second most commonly isolated species in the oral cavity followed by *C. krusei* and *C. kefyr/C. glabrata* (20% each). In NP children, *C. krusei* (20%) was the second species followed by *C. tropicalis* (10%) and *C. kefyr*/*C. glabrata* (7.5%). However, these differences were not statistically significant (*p* > 0.05).

**Table 5:** Distribution of different *Candida* species colonized in the oral cavity of children with orofacial clefts and normal palate

*Candida* species	Children with OFCs (*n* = 40)		Children with NP (*n* = 40)		Total (*n* = 80)		*p*
	No.	%	No.	%	No.	%	
*C. albicans* ^*#*^	19	47.5	17	42.5	36	45.0	0.653
*C. tropicalis* ^*#*^	10	25	4	10	14	17.5	0.077
*C. krusei* ^*#*^	8	20	8	20	16	20	1.000
*C. kefyr/C. glabrata* ^#^	8	20	3	7.5	11	13.7	0.105

*OFCs* Orofacial clefts, *NP* Normal palate

^#^Multiple response variables

### *Candida* species and cleft palate types

Table 6 shows that significantly, all (100%) oral swabs from children with CLP showed positive *Candida* colonization compared to 63.6% (*n* = 10) from children with CP (*p* = 0.03) and 47.5% in children with NP (*p* = 0.004). *C. albicans* was prevalent in 50% of patients (*n* = 9) with CLP compared to 27.3% (*n* = 6) in CP children (*p* = 0.030). Regarding *Candida* colonization in CP *versus* NP children, results showed that there was no significant difference in *Candida* growth and distribution between both groups (*p* = 0.347). 

**Table 6:** Distribution of *Candida albicans* and non*-albicans Candida* in different cleft types and normal palate

		No Growth	Growth			*p*
CP (*n* = 22)	Total No. Total %	8 36.4	14 63.6			-
NP (*n* = 40)	Total No. Total %	17 42.5	23 57.5			-
CLP (*n* = 18)	Total No. Total %	0 0	18 100			-

		No Growth	*C. albicans* mono-species cultures	Mixed *C. albicans* and NAC	NAC mono-species cultures	***p***
CP (*n* = 22)	No. %	8 36.4	6 27.3	1 4.5	7 31.8	0.347
NP (*n* = 40)	No. %	17	11 27.5	6 15.0	6 15.0	
		42.5				
CLP (*n* = 18)	No. %	0 0	9 50	2 11.1	7 38.9	0.004*
NP (*n* = 40)	No. %	17 42.5	11 27.5	6 15.0	6 15.0	
CP (*n* = 22)	No. %	8 36.4	6 27.3	1 4.5	7 31.8	0.030*
CLP (*n* = 18)	No. %	0 0	9 50	2 11.1	7 38.9	

*NAC* Non-*albicans Candida,* *NP* Normal palate, *CP* Cleft palate, *CLP* Cleft lip and palate

*Statistically significant at *p* ≤ 0.05

### *Candida* colony counts

The total median (IQR) *Candida* count was significantly higher in children with OFCs 69.0 (410.5) compared with those with NP 1.0 (12.0) (p < 0.001). Similarly, *C. albicans *mono-species cultures median (IQR) demonstrated significantly elevated counts in the OFC group 120.0 (477.0) compared to the NP group 5.0 (10.0) (*p* = 0.001). In contrast, although mixed *C. albicans* and non-albicans *Candida* (NAC) cultures showed higher median (IQR) values in the OFC group 129.0 (138.0) relative to the NP group 33.5 (88.3), this difference did not reach statistical significance (*p* = 0.071). Notably, NAC mono-species cultures median values were also significantly more prevalent in the OFC group 90 (544.5) compared to the NP group 3.5 (44.5), with a statistically significant difference (*p* = 0.023) (Table 7).

**Table 7:** Distribution of the median colony count number of different *Candida* species in children with orofacial clefts and normal palates

Colony counts (CFU/ml)	Children with OFC		Children with NP		Total		*p*
	Median	IQR	Median	IQR	Median	IQR	
Total *Candida* count	69.0	410.5	1.0	12.0	75.7	88.7	< 0.001*
*C. albicans* mono-species culture	120.0	477.0	5.0	10.0	35.0	196.7	0.001*
Mixed *C. albicans* and NAC	129.0	138.0	33.5	88.2	86.0	119.0	0.071
NAC mono-species culture	90.0	544.5	3.5	44.5	23.0	426.7	0.023*

*IQR* Interquartile range, *NP* Normal palate, *CFU/ml* colony-forming unit per milliliter, *OFCs* Orofacial clefts

* Statistically significant at *p* ≤ 0.05

### Phospholipase activity

Phospholipase activity was mostly detected in *C. albicans* strains isolated from both OFCs and NP individuals. All *C. tropicalis* isolates showed negative phospholipase activity (Table 8).

**Table 8:** Phospholipase and hemolysin activity among different *Candida* species in children with orofacial clefts and normal palates

	Children with OFCs		Children with NP		Total		*p*
	No.	%	No.	%	No.	%	
Phosphlipase activity (Pz)							
* C. albicans*^#^ Pz							0.658
• Negative	4	22.2	2	11.8	6	17.1	
• Positive	14	77.8	15	88.2	29	82.9	
* C. tropicalis*^#^ Pz							-
• Negative	10	100.0	4	100.0	14	100.0	
• Positive	0	0.0	0	0.0	0	0.0	
* C. krusei*^#^ Pz							1.000
• Negative	7	87.5	8	100.0	15	93.8	
• Positive	1	12.5	0	0.0	1	6.3	
* C. kefyr/C. glabrata*^#^ Pz							0.273
• Negative	8	100.0	2	66.7	10	90.9	
• Positive	0	0.0	1	33.3	1	9.1	
Hemolysin activity (HI)							
* C. albicans*^#^ HI							0.603
Negative	3	16.7	1	5.9	4	11.4	
Positive	15	83.3	16	94.1	31	88.6	
* C. tropicalis*^#^ HI							1.000
Negative	2	20.0	0	0.0	2	14.3	
Positive	8	80.0	4	100.0	12	85.7	
* C. krusei*^#^ HI							0.315
Negative	2	25.0	5	62.5	7	43.8	
Positive	6	75.0	3	37.5	9	56.3	
* C. kefyr/C. glabrata*^#^ HI							1.000
Negative	3	37.5	1	33.3	4	36.4	
Positive	5	62.5	2	66.7	7	63.6	

*OFCs* Orofacial clefts, *NP* Normal palate

^#^Multiple response variables

### Hemolysin activity

All species isolated from patients with OFCs presented positive hemolytic activity. However, the presence of CP was not significantly associated with hemolysin activity in any of the *Candida* spp. (*p* > 0.05) (Table 8).

## Discussion

This study aimed to address the colonization rate of *Candida* spp. found in the oral cavity of children with OFCs compared to children with NP. As far as literature has been searched, whether clefted palates predisposes patients to colonization by specific *Candida* spp. has not been extensively studied. This point would be important to study since *Candida* spp. vary in their pathogenicity and response to antifungals. The importance of determining the oral carriage rate of *Candida* spp. and assessing their virulence factors is to assess the need for administering antifungal therapy to cleft palate patients and identify if specific types of clefts are more prone to colonization than others. Moreover, determining whether enzyme production by different *Candida* spp. is accentuated among OFCs patients would help understand the pathogenesis of oral candidiasis among this group of patients.

*Candida* overgrowth in the oral cavity might cause pain and discomfort, change in taste, eating and swallowing difficulties, dysphagia (if spread to the esophagus) and consequently inadequate nutrition. In patients with compromised immune systems, infection can spread into the bloodstream, causing candidemia or to the upper gastrointestinal tract, causing severe infections with increased morbidity and mortality [26]. In the present study, no significant association was found between *Candida* oral colonization and any of the following factors: age, sex, bottle feeding and bad oral hygiene in either of the two studied groups, although other studies reported significant differences (age [27], sex [28], bottle feeding [29] and bad oral hygiene [18]).

The findings of the present study led to the rejection of the null hypothesis, as a statistically significant difference in oral *Candida* colonization was observed between children with OFCs and those with NP. The present study reported higher oral colonization rates by *Candida* spp. in patients with OFCs (80%) compared to children with NP (57.5%). Significantly higher colony counts were also recovered from OFC patients than from NP children median (IQR) values of 69.0 (410.5) *versus* 1.0 (88.75). This was in concordance with the higher colonization rates reported in other studies comparing OFC children to NP children [8, 17, 30]. The significantly higher colonization prevalence and colony counts among OFC children puts them at higher risk of *Candida* infection, whether locally in their oral cavity or systemically through blood spread. However, the present study did not follow up *Candida* colonization to investigate any possible further complications. Therefore, longitudinal monitoring is recommended in future research to better understand the progression and clinical impact of colonization over time. Batista et al. reported an association between oral *Candida* colonization and systemic infection in high-risk patients; however, such outcomes represent severe clinical scenarios that are not representative of typical *Candida* colonization [31]. In contrast, *Candida* colonization is more frequently associated with localized conditions, including denture-related stomatitis, where biofilm formation and local factors contribute to mucosal inflammation without systemic spread [32].

Regarding species distribution, the present study reported *C. albicans* as the most frequently encountered species in both groups, with comparable prevalence in children with OFCs (47.5%) and those with NP (42.5%). This finding is in line with previous literature, which has similarly reported *C. albicans* as the predominant oral *Candida* species in both cleft and non-cleft populations [17, 33]. In contrast, variation was observed in the distribution of NAC species. In the present study, *C. tropicalis* emerged as the second most common species in the OFC group (25%), followed by *C. krusei* and *C. kefyr/ C. glabrata* (each 20%). Previous studies have also demonstrated differences in secondary species distribution such as *C. glabrata* [17] and *C. parapsilosis* [33], highlighting inter-study variability in species colonization patterns.

Regarding the mixed *Candida* cultures from patients in both groups, mixed *C. albicans* with other species of NAC were recorded in only 11.3% of children. The colonization frequency by heterogeneous species of *C. albicans* with NAC was lower than that of *C. albicans* (32.5%) or NAC (25%) mono-species cultures. These findings suggest that patients presented more mono-species *Candida* cultures and this might be because *C. albicans* produces enzymes that can inhibit the growth of other species, giving it a competitive edge. It can evade the immune response more effectively than many NAC species, allowing it to proliferate in the oral cavity without significant interference [34]. Identification of NAC is important, since *Candida* spp. are considered as heterogeneous species which differ in their morphology, cell wall composition, size, growth requirements and virulence factors, causing them to have different infection rates and variable immune responses [35]. Additionally, different species of *Candida* have different patterns of resistance to commonly used antifungal treatments, which renders choosing the appropriate antifungal medication a challenge [35, 36].

When comparing the distribution of species based on the type of cleft, results of the present study showed that patients with CLP (100%) had significantly higher colonization than both NP (57.5%) and CP (63.6%) groups. This finding was similar to the results of Rawashdeh et al. [17] who reported that bilateral CLP patients had the highest *Candida* colonization reaching 77.7% and reported that unilateral CLP *Candida* carriage was 57%. These findings might be attributed to the nature of the deformity itself since patients with CLP seem to have larger deformities and greater number of affected oral tissues than patients with other cleft types and thus they are more prone to increased candidal colonization and thus a higher risk of infection [37].

In the present study, the presence of OFCs was not significantly associated with the phospholipase activity in any of the *Candida* spp. (*p* > 0.05). Furthermore, *Candida* spp. in children with OFCs showed positive phospholipase activity at high frequencies (77.8%) in *C. albicans* isolates; but were infrequent in the isolates of *C. krusei* (12.5%) and absent in *C. tropicalis* and *C. kefyr/C. glabrata*. In contrast, previous studies reported lower rates of *C. albicans* phospholipase (36.7%) while similar results were reported for phospholipase activity in NAC species (5.3% for *C. tropicalis* and none for *C. krusei*) [8]. This might be due to variations in strain virulence depending on the geographical location or origin of *Candida* isolates.

The virulence determinants of *Candida* might be overexpressed in OFC cases due to the existing anatomical deformities and tissue lesions [38]. The current study revealed that *C. albicans* showed the highest hemolytic activity (88.6%), followed by *C. tropicalis* (85.7%), *C. kefyr/C.glabrata* (63.6%) and *C. krusei* (56.3%). Singh et al. reported hemolysin production (85.5%) as an important virulence factor in *C. albicans* [39].

In the present work, *Candida* spp. had more hemolysin compared to phospholipase activity. More *Candida* isolates were positive for hemolysin (77.6%) activity than they were for phospholipase (40.8%) activity; however, this difference was not statistically significant. It is probable that among *Candida* spp. isolated from the oral cavity, the more intense production of these enzymes might lead to more severe oral lesions. Additionally, production of these extracellular hydrolytic enzymes appears to be crucial in the development of *Candida* overgrowth, since these enzymes promote host invasion through tissue penetration and adhesion, which may result in a more aggressive infection and clinical picture, furtherly complicating the treatment process [40].

Regarding categorization of colony counts of different *Candida* species, *C. albicans* showed significantly higher colony counts in children with CP than in children with NP. Counts of other *Candida* spp. were not significantly different between OFC and NP children. Since *C. albicans* is known to be the most common and pathogenic *Candida* spp [6]. The higher the counts, the higher the risk for invasiveness and infection. Chabasse et al. study reported a statistically significant association between heavy candiduria (> 10^4^ CFU/ml urine) and a high *Candida* colonization index (> 0.5), indicating extensive colonization across multiple body sites. This suggests that candiduria is not just local — it may reflect systemic colonization, which is a warning sign for possible dissemination [41]. The present study noted that *C. albicans* spp. presented higher count profiles than NAC spp.

The nasal airway is frequently impaired in children with OFCs. Maxillary growth deficits constrict the nasal floor, consequently increasing nasal airway resistance. This usually results in obligatory mouth breathing, which can negatively impact dental and facial development [42]. Parents reported significant mouth breathing in children with OFCs (57.5%) compared to (22.5%) in children with NP. Since OFC children have no synchronization between the oropharyngeal cavity and there is no proper glottic closure for protection of the airways, this therefore increases the risk of choking and aspiration [43]. The present study demonstrated that choking was reported by 26 (65%) of the OFC children’s guardians and by 6 (15%) of NP children’s guardians. Although choking was statistically significant between both study groups, but its association with *Candida* colonization was not.

The colonization of *Candida* spp. encourages the development of biofilms [44], which could potentially impact oral health by causing thrush, also known as pseudomembranous candidiasis [45]. White patches on the oral mucosal surfaces are frequently used as a clinical indicator of thrush. Children with OFCs had a significantly higher prevalence of white patches compared to those with NP (50% *versus* 12%). Moreover, all 100% of NP children with white patches were colonized with *Candida* and 95% of OFC children with white patches were found to be colonized with *Candida*. This might suggest the importance of screening for white patches, especially among OFC children and their use as an indicator for empiric antifungal therapy in such patients.

It is noteworthy that one (5%) clefted child was reported to have white patches yet was not colonized with *Candida*. This indicates that there might be other causes of white patches in the oral mucosa. Conversely, more than half of the children who had no white patches were found to be colonized with *Candida*, indicating that although white patches might be suggestive of *Candida* colonization, however, their absence does not exclude colonization. Other causes of white patches include canker sores, lichen planus, oral cancers or even leukoplakia (an oral white patch that cannot be characterized as any known disease) [46].

In the present study, children with OFCs paid no attention to oral hygiene compared to children with NP, as in most cases (77.8%), the mothers did not perform tooth brushing as recommended. These findings were inconsistent with a previous study [30], which reported that patients with clefts demonstrated good oral hygiene practices (95%) yet still exhibited high rate of oral *Candida* colonization (89.5%), suggesting that oral hygiene practices such as tooth brushing may have a limited impact on reducing oral *Candida* colonization. In the present study, most OFCs children who did not have their teeth brushed (76.2%) were found to have positive *Candida* colonization but showed no statistically significant difference compared to OFCs children with no colonization. The need for strict care regarding oral hygiene in cleft patients is an important concern because maintaining adequate oral hygiene is difficult, which increases their risk of developing oral infections like candidiasis. Several studies reported that patients with cleft had significantly worse oral hygiene than healthy control groups, which might consequently favour greater accumulation of food debris and organic matter and so favour *Candida* colonization [17, 47–52].

In conclusion, this study had several notable strengths. It comprehensively evaluated both *C. albicans* and NAC species, rather than focusing on a single species, providing a broader understanding of oral *Candida* colonization patterns. In addition, two key virulence factors—haemolysin and phospholipase—were assessed, alongside quantitative analysis of *Candida* colony counts, enabling a more integrated evaluation of both pathogenic potential and colonization burden across study groups. However, certain limitations should be acknowledged. The use of CHROMagar allowed identification of only a limited number of *Candida* species, potentially overlooking less prevalent oral species. Therefore, future studies are recommended to utilize more advanced identification systems, such as the VITEK 2 Compact system, to enhance species-level accuracy and detect less common *Candida* species within the oral cavity. Collectively, the findings of this study support the proposed objectives by demonstrating significant differences in oral *Candida* colonization and virulence profiles between patients with OFCs and controls, highlighting the clinical relevance of comprehensive *Candida* assessment in this population.

## Conclusion

This study demonstrates differences in oral *Candida* colonization between children with OFCs and those with NPs, with variation in colonization burden and species distribution between the two groups. The assessment of key virulence factors did not reveal group- or species-specific associations. These findings highlight the importance of comprehensive assessment of oral *Candida* colonization in pediatric populations with OFCs.

## Data Availability

The datasets used and analysed during the current study are available from the corresponding author upon reasonable request.

## Abbreviations

*C. albicans*: *Candida albicans*
*C. glabrata*: *Candida glabrata*
*C. kefyr*: *Candida kefyr*
*C. krusei*: *Candida krusei*
*C. parapsilosis*: *Candida parapsilosis*
*C. tropicalis*: *Candida tropicalis*
CFU/ml: Colony-forming unit per milliliter
CLP: Cleft lip and palate
CP: Cleft palate
HI: Hemolytic index
IQR: Interquartile range
kg: Kilograms
NAC: Non-*albicans* *Candida*
NP: Normal palate
OFCs: Orofacial Clefts
Pz: Phospholipase index
SD: Standard deviation
SDA: Sabouraud dextrose agar
spp.: Species
WHO: World Health Organization
